# Posterior‐chamber phakic implantable collamer lenses with a central port: a review

**DOI:** 10.1111/aos.14599

**Published:** 2020-08-25

**Authors:** Robert Montés‐Micó, Ramón Ruiz‐Mesa, José Luís Rodríguez‐Prats, Pedro Tañá‐Rivero

**Affiliations:** ^1^ Oftalvist Alicante Spain; ^2^ Optics and Optometry and Vision Sciences Department University of Valencia Valencia Spain

**Keywords:** adverse events, implantable collamer lens, intraocular pressure, myopia, phakic, refraction, vault, visual acuity

## Abstract

We aimed to summarize the outcomes reported following the implantation of the V4c implantable collamer lens with a central port (ICL, STAAR Surgical Inc) for myopia correction. A literature search in PubMed, Web of Science and Scopus was carried out to identify publications reporting clinical outcomes of patients who were implanted with the V4c ICL model and had a follow‐up period of at least 6 months. A total of 35 clinical studies published between 2012 and 2020 were included in the present review. A comprehensive analysis of the available data was performed, focusing on visual and refractive outcomes at different time‐points post‐surgery. In addition, adverse events and other parameters such as endothelial cell density, intraocular pressure and vault measurements—which were evaluated in some of the studies—were also compared. This review encompassed a total of 2904 eyes. The outcomes reported in this review lead us to conclude that ICL V4c implantation for myopia correction is a safe and efficient procedure, with stable visual and refractive outcomes and low adverse event rates. The patient’s anterior segment should be thoroughly characterized, and the ICL parameters should be carefully selected so as to achieve good outcomes and avoid complications.

## Introduction

Phakic intraocular lenses have been widely used for the correction of various degrees of refractive errors, including myopic and hyperopic ones alone, for which spherical lenses are used, or combined with astigmatism, which requires toric lenses. Different types of lenses have been used: angle‐supported anterior chamber lenses, iris‐fixated anterior chamber lenses and posterior‐chamber lenses. Differences in design, selection criteria, surgical techniques, outcomes and complications have been reported in different review publications (Güell et al. 2010; Kohnen et al. 2010). Specific attention has been devoted to posterior‐chamber phakic lenses, with review publications focusing on potential complications (Fernandes et al. 2011) and their use in keratoconic eyes (Esteve‐Taboada et al. 2017) or for hyperopia correction (Alshamrani & Alharbi 2019).

One of the most widely‐used and worldwide‐used phakic intraocular lens types is the implantable collamer lens (ICL; STAAR Surgical Inc, Monrovia, CA, USA). This lens has undergone several modifications, from its initial design to the latest one, namely the V4c model, in an attempt to overcome complications and disadvantages (Fernandes et al. 2011). The model, which came onto the market in 2011, incorporates a 0.36‐mm central port (i.e. a hole in the centre) (KS Aquaport), thus making iridectomies or iridotomies unnecessary and allowing for adequate aqueous flow maintaining the normal physiology of the anterior segment. In vitro laboratory studies have shown that the hole‐equipped and non‐hole models provide good and comparable optical quality (Pérez‐Vives et al. 2013; Domínguez‐Vicent et al. 2015); moreover, the effectiveness and safety of this lens have been demonstrated in the framework of clinical studies (Packer 2016; Packer 2018). Taking into account that we can find patients whose follow‐up lasted for about a decade and that there is an increasing number of publications focusing on mid‐ and long‐term outcomes, ‐a factor that should be ranked as one of the most important ones in this type of surgery‐, allthis makes it necessary to carry out a complete and updated analysis of the clinical outcomes reported in the peer‐reviewed literature.

Consequently, the main purpose of the present paper is to provide an updated review of the visual and refractive outcomes, including a detailed analysis of possible adverse events and complications, that the phakic V4c ICL model has yielded in myopia correction procedures, in the context of studies published in peer‐reviewed journals.

## Methods

We explored the following databases for the initial literature review: PubMed (U.S. National Library of Medicine), Web of Science (Thomson Reuters) and Scopus (Elsevier). The search was limited to publications written in English, published in peer‐reviewed journals and focusing on the V4c ICL. The types of studies that we included were prospective, retrospective and comparative. No date restriction was applied to the electronic searches; however, since this model was launched in 2011, all the studies resulting from the search were published subsequently. Bearing in mind that the main objective of this review study was to analyse mid‐ and long‐term outcomes, we only included studies whose follow‐up period was at least six months for all patients. The date of the last electronic search was April 1, 2020. The literature search included a combination of the following keywords: ‘implantable collamer lens’, ‘ICL’, ‘phakic lens’, ‘myopia’, ‘treatment’ and ‘V4c’. Moreover, for each selected study, all its references were also screened to ensure that we would not miss any relevant studies on this topic.

The literature search resulted in 35 articles being identified and subsequently analysed. The oldest paper was published in 2012 and the most recent one in 2020. The following information was extracted from each paper: authors, year of publication, title of the study, journal of publication, sample size (number of eyes and patients), follow‐up time (maximum), age, pre‐ and postoperative spherical equivalent (SE), white‐to‐white (WTW) distance, anterior chamber depth (ACD), keratometry (K), central corneal thickness (CCT), implanted ICL power and size, postoperative uncorrected distance visual acuity (UDVA) and corrected distance visual acuity (CDVA), safety and efficacy indices, predictability (% of eyes within ± 0.50 D and ± 1.00 D of the target refraction), postoperative intraocular pressure (IOP), endothelial cell density (ECD) and percentage loss, vault and number of adverse events or complications. Mean, standard deviation and ranges were included for all parameters analysed, whenever available. Note that for those publications that reported outcomes at different postoperative time‐points we have only considered those values corresponding to the longest follow‐up.

## Results

As mentioned in the methods section, 35 articles meeting the abovementioned criteria resulted from the search. They were published between 2012 and 2020. Table 1 presents a summary of these 35 clinical studies reporting data on the V4c ICL (Shimizu et al. 2012; Alfonso et al. 2013; Lisa et al. 2015; Karandikar et al. 2015; Shimizu et al.2016; Bhandari et al. 2016; Eissa et al. 2016; Chen et al. 2016; Cao et al. 2016a; Cao et al. 2016b; Goukon et al. 2017; Ganesh et al. 2017; Rodríguez‐Una et al. 2017; Pjano et al. 2017; Kamiya et al. 2017; Totsuka et al. 2017; Fernández‐Vigo et al. 2017; Kamiya et al. 2018; Garcia‐de la Rosa et al., 2018; Fernández‐Vega‐Cueto et al. 2018; Yan et al. 2018; Kojima et al.2018; Li et al. 2018; Takahashi et al. 2018; Lee et al. 2018a,b; Alfonso et al. 2019; Rizk et al. 2019; Zhu et al. 2019; Niu et al. 2019; Sachdev et al. 2019; Martínez‐Plaza et al. 2019; Wan et al. 2019; Niu et al. 2020; Chen et al. 2020): authors, maximum follow‐up, number of eyes and patients included, sample mean age, preoperative SE, WTW, ACD, K, CCT and ICL size and power are shown. The outcomes of the 2904 eyes implanted with the V4c ICL model are described and analysed in these studies. Kamiya et al. (2018) had the largest sample of all 35 studies with 351 eyes followed up for one year, and Shimizu et al. (2016) and Alfonso et al. (2019) the longest follow‐up period with five years (26 and 143 eyes, respectively). Regarding age, the mean patient age was 30 years. The age range of the studies was from 18 years in Kamiya et al. (2018) and Zhu et al. (2019) up to 57 years in Kamiya et al. (2018). In fact, this study was the one including the largest age range. Note that Kamiya et al. (2017) (in another study) and Takahashi et al. (2018) had the highest mean patient age, namely 46.1 and 45 years, respectively. The reason behind these high values is that these authors considered the use of ICL as a monovision treatment for presbyopia in older patients.

**Table 1:** Clinical studies reporting data for the V4c ICL with a minimum of 6 months of follow‐up. Values are reported as mean ± standard deviation (range).

Author	Follow‐up	Eyes (Patients)	Age (y)	SE (D)	WTW (mm)	ACD (mm)	K* (D)	CCT (μm)	ICL size (mm)	ICL power (D)
Shimizu et al. (2012)	6 months	20 (20)	31.7 ± 8.0 (23 to 49)	−7.36 ± 2.13 (−3.50 to −11.75)	11.5 ± 0.40 (10.7 to 12.1)	3.13 ± 0.22 (2.80 to 3.59)	43.8 ± 1.8 (40.9 to 47.4)	533.1 ± 34.7 (468 to 600)	NR	NR
Alfonso et al. (2013)	6 months	138 (70)	30.5 ± 4.8 (20 to 41)	−8.73 ± 2.54 (−3.00 to −17.50 sph −0.25 to −3.00 cyl)	11.99 ± 0.44 (11.25 to 13.45)	3.31 ± 0.25 (2.80 to 3.40)	NR	539 ± 35 (448 to 625)	13.16 ± 0.34 (12.6 to 13.7)	−9.52 ± 2.60 (−3.50 to −18)
Lisa et al. (2015)	1 year	147 (80)	30.4 ± 4.8 (20 to 40)	−8.80 ± 2.60 (−2.75 to −17.50 sph; 0 to −3.00 cyl)	11.73 ± 0.37 (11 to 13.45)	3.18 ± 0.24 (2.80 to 3.83)	44.44 ± 1.84 (40 to 48) 43.39 ± 1.61 (39.50 to 46.50)	534 ± 36 (441 to 630)	13.1 ± 0.3 (12.6 to 13.7)	−9.65 ± 2.47 (−3.50 to −18)
Karandikar et al. (2015)	1 year	34 (34)	26.1 ± 3.8 (NR)	−9.24 ± 2.4 (NR)	NR	NR	NR	NR	NR	NR
Cao et al. (2016a)	6 months	63 (32)	30.6 ± 7.9 (21 to 43)	−12.81 ± 3.11 (−5.75 to −17.50)	11.4 ± 0.4 (10.6 to 12.5)	3.14 ± 0.22 (2.80 to 3.85)	NR	515.6 ± 38.6 (419 to 594)	12.7 ± 0.4 (12.1 to 13.7)	−14.14 ± 3.02 (−7 to −18)
Shimizu et al. (2016)	5 years	26 (26)	31.9 ± 7.5 (23 to 49)	−7.54 ± 2.40 (−2.00 to −13.25)	11.5 ± 0.4 (10.7 to 12.1)	3.13 ± 0.20 (2.80 to 3.59)	43.8 ± 1.8 (39.5 to 47.4)	535.0 ± 33.9 (468 to 600)	NR	NR
Bhandari et al. (2016)	9 months	10 (5)	26.1 ± 3.8 (NR)	−9.14 ± 2.4 (NR)	NR	NR	NR	NR	NR	NR
Eissa et al. (2016)	18 months	54 (27)	29 ± 2.3 (NR)	NR (NR)	11.7 ± 0.4 (NR)	NR	NR	NR	12.6 ± 05 (NR)	−6.85 ± 2.30 (NR)
Chen et al. (2016)	6 months	22 (22)	26.5 ± 5.8 (20 to 35)	−9.43 ± 5.01 (−4.75 to −15.75)	11.7 ± 0.8 (10.5 to 12.2)	3.42 ± 0.31 (2.90 to 3.68)	NR	NR	12.4 ± 0.8 (11.0 to 13.0)	NR
Cao et al. (2016b)	6 months	78 (41)	29.1 ± 8.3 (21 to 44)	−12.55 ± 2.98 (−5.75 to −17.50)	11.4 ± 0.4 (10.6 to 12.5)	NR	NR	NR	NR	NR
Goukon et al. (2017)	2 years	34 (34)	32.1 ± 6.6 (23 to 49)	−7.99 ± 2.57 (−3.25 to −11.75)	11.5 ± 0.4 (10.7 to 12.1)	3.14 ± 0.27 (2.80 to 3.81)	43.7 ± 1.7 (41.3 to 47.4)	534.6 ± 33.5 (489 to 600)	NR	NR
Ganesh et al. (2017)	1 year	NR (NR)	26.4 ± 2.4 (NR)	−5.98 ± 1.15 (NR)	NR	NR	NR	NR	NR	NR
Rodríguez‐Una et al. (2017)	2 years	78 (NR)	NR (NR)	NR (NR)	12.4 ± 0.43 (10.76 to 13.65)	3.32 ± 0.24 (2.80 to 3.99)	NR	NR	12.16 ± 0.29 (12.6 to 13.7)	−9.18 ± 2.92 (−2.50 to −18)
Pjano et al. (2017)	1 year	28 (16)	28.2 ± 4.0 (21 to 35)	−9.52 ± 3.69 (NR)	NR	3.48 ± 0.27 (3.09 to 4.16)	NR	NR	NR	NR
Kamiya et al. (2017)	6 months	17 (17)	46.1 ± 4.2 (40 to 53)	−8.67 ± 4.35 (−2.25 to −18.25)	11.7 ± 0.4 (11.0 to 12.6)	3.14 ± 0.28 (2.82 to 3.74)	NR	NR	NR	NR
Totsuka et al. (2017)	6 months	28 (28)	31.1 ± 6.8 (25 to 42)	−7.38 ± 2.26 (−3.25 to −11.80)	NR	NR	NR	NR	NR	NR
Fernández‐Vigo et al. (2017)	2 years	54 (27)	31.2 ± 5.1 (22 to 44)	−8.48 ± 4.03 (sph) (−2.25 to −21.0) −1.56 ± 1.13 (cyl) (0 to −5)	12.1 ± 0.3 (11.4 to 12.7)	3.23 ± 0.28 (2.80 to 3.97)	NR	NR	13.2 ± 0.3 (12.1 to 13.7)	−11.1 ± 2.2 (sph) (−3.5 to −18) 1.3 ± 1.5 (cyl) (0 to 5)
Kamiya et al. (2017)										
Low‐moderate myopia	1 year	57 (57)	34.8 ± 7.4 (20 to 57)	−4.29 ± 1.31 (−0.50 to −5.88)	11.6 ± 0.4 (10.8 to 12.5)	3.08 ± 0.27 (2.80 to 4.02)	43.5 ± 2.2 (37.4 to 51.1)	NR	NR	NR
High myopia		294 (294)	33.6 ± 7.3 (18 to 54)	−10.13 ± 2.64 (−6.00 to −18.63)	11.6 ± 0.4 (10.6 to 12.7)	3.14 ± 0.26 (2.80 to 4.06)	43.9 ± 1.4 (40.1 to 48.3)			
Garcia‐de la Rosa et al. (2018)	1 year	76 (42)	27.4 ± 5.14 (20 to 39)	−11.94 ± 3.51 (−7.50 to −22.88)	11.59 ± 5.6 (10.35 to 12.51)	3.28 ± 0.19 (2.92 to 3.79	NR	521 ± 46.88 (428 to 638)	NR	NR
Fernández‐Vega‐Cueto et al. (2018)	3 years	184 (92)	30.4 ± 5.4 (20 to 45)	−8.30 ± 2.98 (−2.88 to −18.25)	12.0 ± 0.45 (11 to 13.45)	3.32 ± 0.25 (2.80 to 3.91)	NR	534 ± 33 (441 to 601)	13.2 ± 0.3 (12.6 to 13.7)	−9.11 ± 2.83 (−3.5 to −18)
Lee et al. (2018a)	6 months	236 (236)	28.2 ± 5.1 (20 to 44)	−9.19 ± 2.36 (−4.00 to 19.13)	11.46 ± 0.28 (10.85 to 12.80)	3.35 ± 0.20 (2.88 to 3.89)	NR	NR	12.6	−11.2 ± 2.2 (−5.5 to −18)
Lee et al. (2018b)	6 months	52 (52)	26.7 ± 4.6 (19 to 38)	−9.09 ± 2.32 (−5.12 to −16.31)	11.56 ± 0.39 (10.51 to 12.42)	3.35 ± 0.26 (2.86 to 4.00)	44.5 ± 1.5 (41.4 to 48.8) 42.3 ± 1.3 (39.5 to 45)	NR	12.54 ± 0.28 (12.1 to 13.1)	NR
Yan et al. (2018)	2 years	31 (32)	30.8 ± 8.0 (20 to 45)	−14.62 ± 4.29 (−8 to −25.75)	NR	3.15 ± 0.23 (NR)	NR	NR	12.93 ± 0.42 (12.1 to 13.7)	−14.10 ± 2.85 (−8 to −18)
Kojima et al. (2018)	6 months	19 (19)	32.4 ± 5.7 (NR)	−7.32 ± 3.42 (NR)	11.55 ± 0.45 (NR)	3.04 ± 0.29 (NR)	NR	NR	NR	NR
Li et al. (2018)										
Without cysts	1 year	147 (NR)	NR	−12.76 ± 4.00 (sph) −1.78 ± 1.13 (cyl)	NR	3.16 ± 0.24 (NR)	NR	526.0 ± 28.3 (NR)	NR	NR
With cysts		54 (NR)		−13.71 ± 5.01 (sph) −1.44 ± 0.92 (cyl)		3.25 ± 0.19 (NR)		528.6 ± 37.9 (NR)		
Takahashi et al. (2018)	6 months	42 (21)	45.0 ± 3.8 (40 to 53)	−7.37 ± 3.18 (−2.25 to −14.75)	11.6 ± 0.4 (11.0 to 12.3)	3.04 ± 0.22 (2.80 to 3.55)	NR	NR	NR	NR
Alfonso et al. (2019)	5 years	147 (83)	31 ± 24 (22 to 51)	−9.20 ± 3.02 (−3.13 to −18.25)	11.71 ± 0.36 (11 to 13.20)	3.16 ± 0.26 (2.80 to 3.90)	44.51 ± 1.67 (40.75 to 48) 43.43 ± 1.54 (40 to 46.5)	534.0 ± 38.3 (448 to 630)	13.11 ± 0.32 (12.6 to 13.7)	−9.98 ± 2.8 (−3 to −18)
Rizk et al. (2019)	1 year	19 (NR)	26.3 ± 6.6 (NR)	−14.72 ± 3.02 (NR)	NR	3.57 ± 0.19 (NR)	NR	556.6 ± 41.7 (NR)	NR	NR
Zhu et al. (2019)	6 months	128 (65)	26.0 ± 6.7 (18 to 43)	−10.9 ± 2.8 (NR)	NR	NR	NR	NR	NR	NR
Niu et al. (2019)	1−2 years	51 (31)	32.4 ± 6.8 (20 to 42)	−14.03 ± 4.46 (−7.50 to −25.75)	11.67 ± 0.33 (11 to 12.9)	2.74 ± 0.04 (2.65 to 2.79)	NR	NR	12.58 ± 0.31 (12.1 to 13.7)	NR
Sachdev et al. (2019)	1 year	203 (NR)	24 ± NR (22 to 28)	−8.37 ± NR (−6.37 to −12.37)	11.7 ± NR (11.5 to 11.95)	3.19 ± NR (469 to 508)	45.3 ± NR (44.2 to 46) 44 ± NR (43.2 to 44.8)	NR	NR	NR
Martínez‐Plaza et al. (2019)	6–46 months	30 (30)	32.4 ± 5.8 (NR)	−7.06 ± 4.04 (NR)	NR	NR	NR	NR	NR	NR
Wan et al. (2019)										
SE (≤−6D)	6 months	137 (137) 27 (NR)	27.6 ± 6.1 (21 to 41)	−5.33 ± 0.83 (−2.63 to −6)	NR	NR	NR	NR	NR	NR
SE (−6.13 to −9D)		29 (NR)	26.3 ± 4.0 (21 to 36)	−8.01 ± 0.82 (−6.13 to −9)						
SE (−9.13 to −12D)		54 (NR)	28.8 ± 7.0 (21 to 47)	−10.67 ± 0.94 (−9.13 to −12)						
SE (−12.13 to −18D)		27 (NR)	32.9 ± 9.6 (21 to 50)	−14.01 ± 1.47 (−12.13 to −17.90)						
Niu et al. (2020)	1 year	39 (20)	27.3 ± 5.4 (NR)	−7.54 ± 1.07 (NR)	NR	NR	NR	514.8 ± 42.1 (NR)	NR	NR
Chen et al. (2020)	1 year	26 (26)	29.3 ± 6.6 (18 to 44)	−12.48 ± 3.78 (−6.63 to −26.63)	NR	NR	NR	NR	NR	NR

ACD = anterior chamber depth, CCT = central corneal thickness, D = dioptres, ICL = implantable collamer lens, K = keratometry, NR = not reported, SE = preoperative spherical equivalent, WTW = White‐to‐white. * one value means average keratometry, two values indicate steep and flat keratometry.

The average preoperative SE across all the studies was −9.55 D, but as for individual SE values it ranged from −0.50 D (Kamiya et al. 2018) to −25.75 D (Yan et al. 2018). Rizk et al. (2019) showed the highest mean SE value (−14.72 D), but unfortunately no range was provided for this sample. WTW and ACD parameters were not reported in 13 and 11 papers, respectively. Mean WTW and ACD across those studies that did assess these parameters were 11.7 mm and 3.20 mm, respectively. Chen et al. (2016) reported the smallest WTW value (10.5 mm) and Rodríguez‐Una et al. (2017) the largest WTW value (13.65 mm). As for ACD, we found values ranging from 2.65 mm, as reported by Niu et al. (2019), to 4.16 mm given by Pjano et al. (2017). Niu et al. (2019) specifically evaluated the use of ICL in patients with shallow ACD (i.e. ACD ranging from 2.65 to 2.79 mm). As for K and CCT values, they were less frequently reported; K ranged from 37.40 to 51.1 D (Kamiya et al. 2018), and mean CCT was 419 μm (Cao et al. 2016a) and 638 μm (Garcia‐de la Rosa et al. 2018). Both groups of values cover a broad range of corneal K and thickness.

Mean ICL size across all studies was 12.8 mm and ranged from 12.1 to 13.7 mm. Note that the lens is manufactured in 4 sizes: 12.1, 12.6, 13.2 and 13.7 mm, and the use of one size or another depends on the anterior segment characteristics, which may vary as a function of the ethnicity of the patient (Chang & Meau 2007; Qin et al. 2012; Lee et al. 2015; Chansangpetch et al. 2018). In relation to ICL power, the overall mean value was −10.48 D, with Cao et al.’s (2016a) being the study reporting the highest mean ICL power (−14.14 D) and Eissa et al.’s (2016) reporting the lowest one (−6.85 D). If we analyse the ranges for the studies reporting this parameter, the smallest value was reported by Rodríguez‐Una et al. (2017) with a value of −2.50 D; the highest value of −18 D was reported in several studies.

Table 2 summarizes the visual outcomes reported in each clinical study included in the present review. Note that some of them did not report visual acuity values (UDVA and CDVA) or safety and efficacy indices; in some cases, it was due to the fact that they focused on other parameters instead, such as IOP or endothelial cell changes. Table 3 details the postoperative refractive outcomes reported, the predictability of the procedure (percentage of eyes ± 0.50/1.00 D) and the mean SE. Table 4 describes in detail the outcomes for IOP, ECD and vault for the different studies when data are available. Table 5 summarizes the most important and prevalent adverse events and complications that have been reported in the different publications analysed.

**Table 2:** Visual postoperative outcomes for the clinical studies considered in the review. Values are reported as mean ± standard deviation.

Author	UDVA (logMAR)	CDVA (logMAR)	Safety Index	Efficacy Index
Shimizu et al. (2012)	−0.20 ± 0.12	−0.25 ± 0.06	1.13 ± 0.24	1.03 ± 0.30
Alfonso et al. (2013)	0.009 ± 0.062	−0.015 ± 0.032	1.01	1.00
Lisa et al. (2015)	0.028 ± 0.055	0.003 ± 0.013	1.04	1.00
Karandikar et al. (2015)	NR	NR	1.15	1.6
Cao et al. (2016a)	0.118 ± 0.096	0.018 ± 0.035	1.42 ± 0.34	1.11 ± 0.19
Shimizu et al. (2016)	−0.17 ± 0.14	−0.24 ± 0.08	NR	NR
Bhandari et al. (2016)	NR	NR	1.14	1.5
Eissa et al. (2016)	NR	NR	NR	NR
Chen et al. (2016)	NR	NR	NR	NR
Cao et al. (2016b)	0.13 ± 0.10	0.05 ± 0.06	NR	NR
Goukon et al. (2017)	NR	NR	NR	NR
Ganesh et al. (2017)	−0.022 ± 0.021	−0.071 ± 0.079	1.24	1.12
Rodríguez‐Una et al. (2017)	NR	NR	NR	NR
Pjano et al. (2017)	0.76 ± 0.16*	0.79 ± 0.14*	1.25	1.2
Kamiya et al. (2017)	−0.04 ± 0.18	−0.19 ± 0.09	NR	NR
Totsuka et al. (2017)	NR	NR	NR	NR
Fernández‐Vigo et al. (2017)	0.02 ± 0.10	0.01 ± 0.09	NR	NR
Kamiya et al. (2017)				
Low‐moderate myopia	−0.17 ± 0.14	−0.21 ± 0.10	NR	NR
High myopia	−0.16 ± 0.09	−0.21 ± 0.08	NR	NR
Garcia‐de la Rosa et al. (2018)	0.12 ± 0.12	0.05 ± 0.08	NR	NR
Fernández‐Vega‐Cueto et al. (2018)	0.08 ± 0.12	0.01 ± 0.04	1.03	0.90
Lee et al. (2018a)	NR	NR	NR	NR
Lee et al. (2018b)	NR	NR	1.38 ± 0.22	1.35 ± 0.19
Yan et al. (2018)	0.84 ± 0.28*	1.00 ± 0.27*	1.24 ± 0.26	1.03 ± 0.23
Kojima et al. (2018)	−0.23 ± 0.09	−0.25 ± 0.07	1.21 ± 0.20	1.16 ± 0.22
Li et al. (2018)	NR	NR	NR	NR
Takahashi et al. (2018)**	−0.03 ± 0.20	−0.19 ± 0.08	NR	NR
Alfonso et al. (2019)	0.13 ± 0.18	0.02 ± 0.08	1.09 ± 0.36	0.87 ± 0.26
Rizk et al. (2019)	0.33 ± 0.20	0.15 ± 0.10	1.67	1.25
Zhu et al. (2019)	NR	NR	NR	NR
Niu et al. (2019)	0.89 ± 0.30*	1.00 ± 0.27*	1.33 ± 0.60	1.14 ± 0.54
Sachdev et al. (2019)	0 ± NR	0 ± NR	NR	NR
Martínez‐Plaza et al. (2019)	−0.08 ± 0.07	−0.09 ± 0.07	1.13	1.12
Wan et al. (2019)				
SE (≤−6D)	0.02 ± 0.04	0.00 ± 0.00	1.02 ± 0.06	0.98 ± 0.10
SE (−6.13 to −9D)	0.03 ± 0.07	−0.01 ± 0.02	1.02 ± 0.07	0.96 ± 0.14
SE (−9.13 to −12D)	0.02 ± 0.05	0.00 ± 0.03	1.04 ± 0.08	1.01 ± 0.13
SE (−12.13 to −18D)	0.09 ± 0.12	0.01 ± 0.02	1.23 ± 0.20	1.03 ± 0.24
Niu et al. (2020)	−0.10 ± 0.05	−0.12 ± 0.06	1.11 ± 0.15	1.06 ± 0.15
Chen et al. (2020)	NR	NR	1.19 ± 0.23	1.04 ± 0.27

CDVA = corrected distance visual acuity, NR = not reported, UDVA = uncorrected distance visual acuity.

* Visual acuity in Snellen decimal; ** intentional undercorrection

**Table 3:** Refractive postoperative outcomes for the clinical studies considered in the review. Values are reported as mean ± standard deviation.

Author	% ±0.50D	% ±1.00D	SE (D)
Shimizu et al. (2012)	95	100	0.01 ± 0.29
Alfonso et al. (2013)	98.55	100	−0.03 ± 0.19
Lisa et al. (2015)	93.9	100	−0.14 ± 0.26
Karandikar et al. (2015)	57.12	98.12	−0.19 ± 1.18
Cao et al. (2016a)	96.8	100	−0.05 ± 0.27
Shimizu et al. (2016)	88	96	−0.15
Bhandari et al. (2016)	NR	NR	−0.2 ± 1.18
Eissa et al. (2016)	NR	NR	NR
Chen et al. (2016)	NR	NR	NR
Cao et al. (2016b)	NR	NR	−0.07 ± 0.29
Goukon et al. (2017)	NR	NR	NR
Ganesh et al. (2017)	90	100	−0.164 ± 0.20
Rodríguez‐Una et al. (2017)	NR	NR	NR
Pjano et al. (2017)	NR	NR	−0.21 ± 0.27
Kamiya et al. (2017)	100	100	−0.08 ± 0.17*
Totsuka et al. (2017)	NR	NR	NR
Fernández‐Vigo et al. (2017)	NR	NR	−0.02 ± 0.44 (sph) −0.11 ± 0.37 (cyl)
Kamiya et al. (2017)			
Low‐moderate myopia	93	98	−0.12
High myopia	94	99	0.02
Garcia‐de la Rosa et al. (2018)	85	86	−0.06 ± 0.77
Fernández‐Vega‐Cueto et al. (2018)	74.5	91.8	−0.37 ± 0.47
Lee et al. (2018a)	NR	NR	NR
Lee et al. (2018b)	88	100	0.11 ± NR
Yan et al. (2018)	79	98	−0.90 ± 0.95
Kojima et al. (2018)	94.7	100	0.05 ± 0.07
Li et al. (2018)	NR	NR	NR
Takahashi et al. (2018)	100	100	NR
Alfonso et al. (2019)	67.4	90.1	−0.44 ± 0.47
Rizk et al. (2019)	NR	78.9	−0.78 ± 0.70
Zhu et al. (2019)	NR	NR	NR
Niu et al. (2019)	69	92	−0.67 ± 1.29
Sachdev et al. (2019)	94	96	−0.12 ± NR
Martínez‐Plaza et al. (2019)	NR	NR	0.00 ± 0.20
Wan et al. (2019)			
SE (≤‐6D)	96	100	−0.01 ± 0.24**
SE (−6.13 to −9D)	100	100	−0.03 ± 0.24**
SE (−9.13 to −12D)	100	100	0.03 ± 0.33**
SE (−12.13 to −18D)	81	96	0.00 ± 0.44**
Niu et al. (2020)	90	100	0.07 ± 0.23
Chen et al. (2020)	NR	NR	−0.36 ± 0.98

NR = not reported, SE = spherical equivalent. * dominant eye; ** SE change between 1 week and 6 months.

**Table 4:** Intraocular pressure (IOP), endothelial cell density (ECD) and vault for the clinical studies considered in the review. Values are reported as mean ± standard deviation (range).

Author	IOP (mmHg) (eyes> 21)	ECD (cell/mm^2^) (% loss)	Vault (μm)
Shimizu et al. (2012)	13.0 ± 3.0 (0)	2720 ± 268 (2.8)	NR
Alfonso et al. (2013)	12.4 ± 1.5 (0> 20)	2533 (8.5)	482.7 ± 210.5 (90 to 970)
Lisa et al. (2015)	12.4 ± 1.4 (0> 20)	2650 ± 438 (1.7)	405.5 ± 184.7 (100 to 980)
Karandikar et al. (2015)	19.1 ± 1.3NR	NR (7.1)	628.2 ± 300.1 (NR)
Cao et al. (2016a)	15.3 ± 2.0 (0)	2648 ± 317 (2)	505.2 ± 258.9 (120 to 990)
Shimizu et al. (2016)	13.6 ± NR (0)	2799 (0.5 ± 5.4)	NR
Bhandari et al. (2016)	19.9NR	NR (6.1)	612 ± 251.14 (NR)
Eissa et al. (2016)	16.07 ± 4.13 (0)	NR	637 ± 125 (NR)
Chen et al. (2016)	16.0 ± 2.2 (0)	NR	542.8 ± 45.3 (NR)
Cao et al. (2016b)	14.9 ± 2.0 (0)	2633 ± 310 (2.0)	499.7 ± 244.3 (120 to 980)
Goukon et al. (2017)	NR	2806 ± 248 (0.3)	NR
Ganesh et al. (2017)	NR	2808 ± 315 (9.0)	NR
Rodríguez‐Una et al. (2017)	12.7 ± 1.1 (0)	NR	369.9 ± 191.0 (0 to 980)
Pjano et al. (2017)	14.96 ± 1.7 NR	2512 ± 127 (5.5)	NR
Kamiya et al. (2017)	NR (0> 22)	NR	NR
Totsuka et al. (2017)	NR	NR	382.1 ± 176.5 (NR)
Fernández‐Vigo et al. (2017)	15.5 ± 2.11 (0)	2480.8 ± 214.3 (5.9)	458.3 ± 258.4 (NR)
Kamiya et al. (2017)			
Low‐moderate myopia	13.1 (0)	NR (0.1)	NR
High myopia	13.6 (0)	NR (0.1)	NR
Garcia‐de la Rosa et al. (2018)	NR	NR	449 ± 180 (NR)
Fernández‐Vega‐Cueto et al. (2018)	12.8 ± 1.7 (0)	2663 ± 366 (2.88)	349 ± 165 (NR)
Lee et al. (2018a)	NR	NR	519 ± 112.8 (250 to 740)
Lee et al. (2018b)	NR	NR	570 ± 150 (310 to 880)
Yan et al. (2018)	15.86 ± 4.11 (NR)	3246 ± 522 (0.15)	449 ± 167 (NR)
Kojima et al. (2018)	NR	NR	NR
Li et al. (2018)			
Without cysts	16.44 ± 1.98 (NR)	NR (NR)	610 ± 230 (NR)
With cysts	16.78 ± 2.35 (NR)	NR (NR)	510 ± 220 (NR)
Takahashi et al. (2018)	NR (0)	NR	NR
Alfonso et al. (2019)	13.00 ± 2.03 (0)	2645 ± 359 (0.43)	340 ± 163 (NR)
Rizk et al. (2019)	15.64 ± 1.13	NR	NR
Zhu et al. (2019)			
Before dilation	NR	NR	462.5 ± 162.7
After dilation	NR	NR	508.5 ± 162.6
Niu et al. (2019)	15.15 ± 2.57 (0)	2963.6 ± 396.1 (8.38)	380.0 ± 152.8 (90 to 700)
Sachdev et al. (2019)	NR	NR	NR
Martínez‐Plaza et al. (2019)	15.1 ± 2.2 (NR)	NR	428.1 ± 234.1 (NR)
Wan et al. (2019)	NR	NR	NR
Niu et al. (2020)	14.65 ± 2.22 (NR)	2597.03 ± 235.99 (1.67)	581.03 ± 199.87 (220 to 1180)
Chen et al. (2020)	15.52 ± 2.87 (NR)	3261.4 ± 355.1 (0.35)	NR

NR = not reported.

**Table 5:** Adverse events/complications for the clinical studies considered in the review.

Author	Adverse events/complications
Karandikar et al. (2015)	1 eye had anterior subcapsular opacity (2.94%). 2 eyes had rotation> 30° that required re‐rotation surgery. Glare and haloes in 24% and 27% of eyes, respectively.
Bhandari et al. (2016)	Anterior subcapsular opacities present in 3.14% of eyes. 1 eye had rotation> 30° that required re‐rotation surgery. Glare and haloes in 23% and 25% of eyes, respectively
Ganesh et al. (2017)	3 eyes required lens exchange due to frequent rotation by> 30° and excessive high vault
Pjano et al. (2017)	1 eye (3.57%) had retinal detachment 3 months after the ICL implantation. 1 eye required lens exchange with a bigger lens due to rotation
Fernández‐Vigo et al. (2017)	1 eye with mild anterior subcapsular cataract
Kamiya et al. (2017)	
Low‐moderate myopia	Glare and haloes in 5 eyes (8.7%)
High myopia	Glare and haloes in 7 eyes (2.4%). 2 eyes (0.7%) required ICL exchange due to incorrect initial sizing or power. 1 eye (0.3%) developed significant axis rotation (≥30°). 1 eye (0.3%) developed iritis 1 eye (0.3%) required LASIK due to undercorrection.
Fernández‐Vega‐Cueto et al. (2018)	2 patients required re‐rotation surgery due to high vault (>1200 μm) 2 patients required LASIK for refractive error correction
Kojima et al. (2018)	1 patient showed extreme high vault and both ICLs were rotated 90° at fixed perpendicularly decreasing the vault.
Rizk et al. (2019)	4 eyes with pigment dispersion and 1 eye with anterior lens opacity
Sachdev et al. (2019)	1 eye with visually significant cataract

## Discussion

### Visual and refractive outcomes

If we take a closer look at the UDVA and CDVA values (Table 2) we may observe that these values were about 0 logMAR or even better (i.e. negative values) in some cases (Shimizu et al. 2012; Alfonso et al. 2013; Shimizu et al. 2016; Ganesh et al. 2017; Kamiya et al. 2017; Kamiya et al. 2018; Kojima et al. 2018; Takahashi et al. 2018; Martínez‐Plaza et al. 2019; Niu et al. 2020). It should be noted that a couple of studies reported worse values for UDVA and CDVA than the average: Pjano et al. (2017) about 0.8 Snellen decimal and Rizk et al. (2019) about 0.2 logMAR. However, a detailed analysis of these studies reveals that the preoperative values of their patients were low and, consequently, the use of these lenses should be considered successful because postoperative values were equal or better than their preoperative counterparts. This is corroborated by their corresponding safety and efficacy indices: 1.25 and 1.2, and 1.67 and 1.25, respectively. In general, both indices for all the studies were about 1 or higher (in some cases > 1.2), thus confirming the safety and the efficacy of this procedure. However, it is interesting to note that only two studies showed an efficacy index lower than 1, about 0.9 (Fernández‐Vega‐Cueto et al. 2018; Alfonso et al. 2019). Both studies analysed long‐term outcomes: Alfonso et al. (2019) with five years of follow‐up and Fernández‐Vega‐Cueto et al. (2018) with three years of follow‐up. It seems that studies with longer follow‐up periods show worse efficacy (no other studies included in the review with more than one year of follow‐up reported this index). We should take into account that the efficacy index considers postoperative UDVA; hence, if there is an uncorrected refractive error after the surgery this is going to have a direct impact upon its value (see Table 3 for postoperative SE). Consequently, it is plausible than the increase in myopia (progression) with time may be the source of this postoperative refractive error—and the resulting low UDVA and efficacy—and not any other source associated with the ICL implantation procedure. Therefore, those studies with long follow‐up periods and younger patients, where myopia may not be stabilized yet, will probably report lower efficacy indices. Needless to say, this fact will also affect any other refractive surgery procedure, not only phakic intraocular lens implantation.

If we consider possible differences across studies as a function of the preoperative SE or if the amount of myopia may affect the visual outcomes, we should state that there are no differences. Kamiya et al. (2018) analysed low/moderate and high myopia cases separately and the UDVA and CDVA outcomes were similar, ranging from −0.16 to −0.21 logMAR in both groups. Wan et al. (2019) broke down their cases into four groups and, similarly to Kamiya et al. (2018), their values were comparable for visual acuity and indices (see Table 2). In fact, both indices were even higher for the group of eyes having SE>−12 D. Then, the amount of SE to be corrected seems not to be a factor affecting postoperative visual outcomes.

Table 3 details the refractive outcomes reported. Almost all the studies reported that 100% of the eyes achieved ± 1.00 D. Eye groups showing high preoperative SE showed similar percentage values than those having lower SE values. For example, Kamiya et al. (2018) reported 98% and 99% of the eyes in the low/moderate and high myopia groups, respectively. Wan et al. (2019) showed 100% of eyes ± 1.00 D except for eyes with SE> 12 D with 96%. For ± 0.50 D, the percentages varied considerably across studies. For instance, Karandikar et al. (2015) obtained 57.2%, Alfonso et al. (2019) 67.4%, Niu et al. (2019) 69% and Fernández‐Vega‐Cueto et al. (2018) 74.5% of the eyes. On the contrary, other studies reported values close to 100%. As we have previously indicated, those studies with large follow‐ups may be affected by potential myopia progression occurring with time. This correlates well with the relationship between follow‐up time and postoperative SE reported in these studies: Alfonso et al. (2019), Fernández‐Vega‐Cueto et al. (2018) and Niu et al. (2019) followed up their patients for five, three and one to two years, respectively, and their corresponding mean postoperative SE was −0.44, −0.37 D and −0.67 D, respectively. The other study with five years of follow‐up was published by Shimizu et al. (2016): there, 88% of the eyes had a postoperative SE within the ± 0.50 D range, the mean value being −0.19 D. In contrast, we cannot explain the low percentage reported by Karandikar et al. (2015); perhaps other factors, such as incorrect IOL power calculation, were the reason behind this poor predictability. Overall, the overall mean postoperative SE (i.e. all the studies included) is less than a quarter of a dioptre (−0.19 D), which shows the procedure’s good predictability. Large values are related to studies with longer follow‐ups (Fernández‐Vega‐Cueto et al. 2018; Yan et al. 2018; Alfonso et al. 2019; Niu et al. 2019); see Table 3 for detailed values, but they are all <−1.00 D. The predictability of the procedure is also good as a function of the amount of myopia to be corrected. The findings of Kamiya et al. (2018) and Wan et al. (2019) confirm this: their values ranged between 0 and −0.12 D in eyes with preoperative SE myopia of up to −18.63 D. In fact, Kamiya et al. (2018) showed a lower value for the high myopia group than for the low/moderate myopia group (0.02 D versus −0.12 D, respectively). Note that the values reported by Wan et al. (2019) indicate the SE change between one week and six months.

High mean power values are to be expected for this treatment since this procedure is usually carried out in eyes where other corneal laser refractive surgeries, such as photorefractive keratectomy or laser in situ keratomileusis, are contraindicated due to the large amount of SE. Recent comparative studies on high myopia cases support the use of ICL versus small‐incision lenticule extraction (Qin et al. 2019; Siedlecki et al. 2020). However, note that the lowest ranges for the ICL power in some studies are quite low, indicating that this procedure is also considered, as alternative to other laser surgeries in spite of them not being contraindicated. Several studies support the use of ICL versus other laser treatments also in low myopia cases (Sanders and Vukich 2006; Kamiya et al. 2012). Kamiya et al. (2018) and Wan et al. (2019) analysed the outcomes as a function of the level of myopia and they included a group with low SE values (low/moderate group: from −0.50 to −5.88 D, and ≤‐6 D group: from −2.63 to −6 D, respectively), but unfortunately no ICL powers were reported in their respective studies. Notwithstanding, it is expected that low‐powered ICL was used to correct small degrees of myopia, showing the use of these lenses in low myopic eyes.

Visual and refractive outcomes confirm the efficacy, safety and predictability of the use of the V4c ICL model for myopia correction with long follow‐ups.

### Intraocular pressure, endothelial cell density and vault

This section analyses the outcomes for IOP, ECD and vault (Table 4). Consequences of inadequate IOP, ECD or vault values are described in the adverse events subsection.

#### Intraocular pressure

IOP is one of the most important parameters that should be evaluated in patients implanted with this lens. As mentioned above, the central port facilitates aqueous flow, which helps keep IOP at appropriate levels. It has been indicated that laser iridotomy is unnecessary from the viewpoint of theoretical aqueous circulation in the presence of a central hole (Kawamorita et al. 2017). Thus, postoperative IOP values should be carefully analysed. IOP column of Table 4 indicates the mean postoperative values reported, and whether or not any eye showed an IOP value > 21 mmHg. Note that some studies did not report IOP values, which in our opinion should be mandatory when assessing patients that have been implanted with this lens. In this we can observe that no studies showed a mean value higher than 20 mmHg; in fact, the overall mean value (i.e. across all the studies) was 14.9 mmHg. Only Karandikar et al. (2015) and Bhandari et al. (2016) reported values as high as 19.1 and 19.9 mmHg, respectively. No information about CCT or K in these groups of patients is published (see Table 1), which makes it impossible to correlate high CCT or K values with high IOP measurements. In both studies, the sample size was low: 34 and 10 eyes, respectively. In contrast, those studies with the largest samples and the longest follow‐up periods showed lower IOP values: Kamiya et al. (2018) (with 57 and 294 eyes and one year of follow‐up) showed mean values of 13.1 and 13.6 mmHg, respectively, whereas Shimizu et al. (2016) and Alfonso et al. (2019) (with 26 and 147 eyes, respectively) at five years of follow‐up, reported a mean IOP of 13.6 and 13 mmHg, respectively. Shimizu et al. (2016) showed that IOP did not change significantly during the five years of follow‐up (p = 0.53), and, more specifically, Alfonso et al. (2019) reported that most eyes had no IOP change or showed a change within ± 2 mmHg in 83.7% and 80.1% at one and five years, respectively. These studies with long‐term follow‐up demonstrate that there is no significant variation of IOP over time. As far as we are aware, only one study (not included in this review due to the short follow‐up) reported a case of pupillary block with the V4c model (Senthil et al. 2016). This case has also been reported by Grover et al. (2017). The authors explained that this eye showed a very high IOP on day one post‐surgery due to the central hole being blocked by the retained viscoelastic behind the ICL, which was solved with surgery to clear the viscoelastic. As indicated, the central hole offers surgical advantages over non‐hole ICL models since no preoperative iridotomy or intraoperative iridectomy is necessary to prevent IOP increase related to pupillary block or chronic pigment dispersion (Fernandes et al. 2011). This is supported by Shimizu et al. (2016), who carried out a comparative study in patients implanted with a hole‐equipped ICL in one eye and a non‐hole ICL in the other, and showed comparative values of IOP between eyes during the whole follow‐up period of 5 years.

The good outcomes reported in the different series discussed support the proper operation of the central hole in the dynamics of the humour to simplify the surgery.

#### Endothelial cells

The mean postoperative ECD value reported varied considerably depending on patient mean age and follow‐up duration; it ranged between 2400 and 3200 cells/mm^2^ (see Tables 1 and 4). Our main interest was the percentage loss that was reported in each series and those studies with longer follow‐ups; this parameter also varied across the studies. We consider that the largest loss occurs during the early postoperative period, and the surgical procedure is the main cause of this loss (surgeon variable‐dependent), and that the loss tends to achieve a stable state (or with lower rates of loss) after that period. This is in good agreement with what is shown in the studies included in this review: for example, Alfonso et al. (2013), Bhandari et al. (2016), Ganesh et al. (2017), Pjano et al. (2017) and Niu et al. (2020) showed ECD loss values ranging between 5.5% and 8.5% in short periods of follow‐up. In contrast, longer follow‐up studies reported lower values: 2.88% (Fernández‐Vega‐Cueto et al. 2018) at three years, 0.5% (Shimizu et al. 2016) and 0.43% (Alfonso et al. 2019) at 5 years. These values are better compared with studies where the non‐hole ICL was implanted: 6.1% (Pineda‐Fernández et al. 2004) at three years and 3.7% (Kamiya et al. 2009) at four years after ICL implantation. Other authors observed a continuous loss, at a rate of 2% to 3% per year over the first three years (Edelhauser et al. 2004), considering the surgical procedure the cause of the initial loss, whereas further decreases during later periods are assumed to be due to natural cell loss (Bourne et al. 1997). The expected physiologic cell loss is approximately 0.6% per year (Bourne et al. 1997; Edelhauser et al. 2004). Two comparative studies between both ICL models (with and without the central hole) concluded that neither lens induced a significant change postoperatively. Goukon et al. (2017) at two years found a cell loss of 0.3% and 1.1% with and without the hole, respectively, while Shimizu et al. (2016) had similar values at five years: 0.5% and 1.2%, respectively.

Thus, taking into account the values reported in different studies, the use of this lens might induce considerable ECD surgery‐related endothelial cell loss in the short postoperative period (similarly to early models), similar to what is expected for natural ageing over long periods.

#### Vault

Similarly to other parameters, vault also varied broadly across studies (see detailed values in Table 4). The overall mean value is 486 µm, but it ranges from 0 (Rodríguez‐Una et al. 2017) at two years to 1180 µm (Niu et al. 2020) at one year of follow‐up). A proper selection of the ICL size is extremely important in order to avoid under or over‐estimation of its length, which may cause low or high vault. A low vault (<250 µm) increases the risk of cataract formation, and a high vault (>750 µm) increases the risk of angle closure, pupillary block or pigment dispersion glaucoma (Fernandes et al. 2011). No mean value for each of the individual studies was found to be outside of this range, and values varied from 340 µm (Alfonso et al. 2019) to 637 µm (Eissa et al. 2016).

We consider that the mean values reported in the different studies are adequate to avoid postoperative adverse events. However, we want to point out that there is some variability within the values. This may be due to several factors, such as the size of the selected ICL, the time after surgery when vault was measured and also the age of the patient all deserve a thorough discussion. First, ICL size depends on the ciliary sulcus diameter and its direct measurement should be the most appropriate procedure to pick the lens size. This may be done using high‐resolution ultrasound biomicroscopy (UBM), which has been shown to provide more ideal ICL vault than conventional WTW (Choi et al. 2007). Notwithstanding, although UBM has facilitated the measurement of this distance and it use for vault predictability (Reinstein et al. 2013), it is not very widespread in clinical practice, possibly due to its invasiveness and the time required for the measurement. Anterior segment optical coherence tomography has also been used to develop ICL sizing formulas (Nakamura et al. 2018; Nakamura et al. 2020). WTW distance, which was used in the different studies analysed in this review, seems to provide a useful measure to estimate ICL size and, on average, an adequate vault. In addition, a meta‐analysis has demonstrated that sulcus‐to‐sulcus and WTW measurement‐based sizing methods result neither in clinical meaningful nor statistically significant differences in vault (Packer 2016). Nam et al. (2017) have suggested the existence of a buffering zone in V4c ICL sizing; a smaller size of this lens should be considered in patients susceptible to overvaulting (such as those with shallow anterior chamber and high dioptric power lenses). Second, there is a continuous reduction in vault over time. It has been published in a follow‐up series of the non‐hole ICL from 6 to 73 months that there is a decrease in vault greater than 20 µm per month during the first six months of the surgery and about 2 µm per month after 36 months of follow‐up (Alfonso et al. 2012a). This is supported, for the hole‐equipped ICL, by the findings of longer follow‐up studies: a longer follow‐up is linked to lower mean vault. Alfonso et al. (2019) showed a value of 340 µm at five years, Fernández‐Vega‐Cueto et al. (2018) had a value of 349 µm at three years, and Rodríguez‐Una et al. (2017), Fernández‐Vigo et al. (2017) and Yan et al. (2018) had values of 369, 458 and 449 µm, respectively, at two years. No comparative studies of vault between hole‐equipped and non‐hole ICLs with long follow‐up have been published, but, in two studies with short follow‐up periods of six months (Cao et al. 2016b) and one year (Kamiya et al. 2015), it has been concluded that the central hole of the ICL did not significantly affect the lens vault and, consequently, the hole‐equipped ICL behaves similarly to the conventional non‐hole ICL. Eissa et al. (2016) suggested that there is a ‘fountain effect’ with the hole ICL that increased the vault, but the comparative studies mentioned before indicate that both lenses perform similarly. Finally, vault correlates negatively with age (Alfonso et al. 2012b). It is well‐known that ACD decreases in the ageing eye due to the thickening of the crystalline lens, at an average of about 24 µm per year (Atchison et al. 2008), and that there is also an age‐related increase in ciliary muscle anteroposterior thickness (Strenk et al. 2010), which might affect ICL position over the years (forward shift of the ICL). Thus, lower vault values are to be expected in older patients compared to younger ones with the same ocular parameters and ICL size. Also, we want to point out that the crystalline lens rise should be also considered as a key factor that may affect postoperative vault, in line with what has been recently suggested (Gonzalez‐Lopez et al. 2019). These authors described how the iris pushes the ICL down and warps it during miosis, to the extent that it adapts to the posterior surface of the iris, thus decreasing the central vault (Gonzalez‐Lopez et al. 2018). They analysed the crystalline lens rise and its relationship with postoperative ICL vault dynamically, concluding that crystalline lens rise should not be ignored in the clinical evaluation of vault.

The vault outcomes reported in the analysed studies remain adequate during follow‐up, thus avoiding postoperative complications related to the ICL position inside the eye.

### Adverse events/complications

This section describes the most important and prevalent adverse events and complications that have been reported in this review. As high as 25 out of the 35 publications analysed in this review did not report any adverse events or complications. Table 5 summarizes all the events reported in the remaining 10 studies.

Note that, except for rare adverse events, cataract formation is the most frequently documented safety concern related to ICL implantation (Fernandes et al. 2011). In fact, the prevalence of cataract formation has been widely studied in the context of the different ICL models, and different studies indicated that it is more common in older patients and in patients with higher myopia (Sanders 2008; Schmidinger et al. 2010; Alfonso et al. 2015). In a study analysing 781 eyes implanted with the V4c ICL model (range 3–24 months), Alfonso et al. (2015) found zero cases of cataractous eyes. Similarly, a meta‐analysis study (Packer 2018) (including data for 1291 eyes implanted with the V4c) described zero incidence of asymptomatic anterior subcapsular cataract formation. Our analysis (see Table 5) indicates that Karandikar et al. (2015), Bhandari et al. (2016), Fernández‐Vigo et al. (2017), Rizk et al. (2019) and Sachdev et al. (2019) reported one eye each with anterior subcapsular opacity [note that Karandikar et al. (2015) and Bhandari et al. (2016) are from the same research group and some criticism should be shown since authors indicated that they have 3.14% of eyes with anterior subcapsular opacities in a sample of 10 eyes (Packer 2018, 2018b)]. This occurred mainly in the early postoperative period, that is at nine months (Bhandari et al. 2016), one year (Karandikar et al. 2015; Rizk et al. 2019; Sachdev et al. 2019) and two years (Fernández‐Vigo et al. 2017)] and may be related to the surgery (i.e. surgical procedure‐related factors, such as the learning curve and the required skill, which are essential) or an inappropriate ICL vault. Cases of anterior subcapsular cataract due to the irrigation technique have been reported (Steinwender et al. 2017). Surgeons should avoid causing trauma to the crystalline lens during ICL implantation. Non‐surgeon‐related factors, such older age and high myopia, entail a risk as well. As mentioned in the introduction, the mean age of the patients undergoing this surgery was 30.0 years (range: 18–57 years), but so far there are no publications analysing in detail the age as an individual factor for patients that are having this ICL model implanted. In highly myopic eyes, the risk is related to the thicker periphery in high‐power lenses, but studies assessing data of patients with low, moderate and high myopia did not report any cases of cataracts (Kamiya et al. 2018; Wan et al. 2019). Taking into account all the samples of the 35 publications (2904 eyes) and five eyes with this complication, we may consider that the prevalence of this event is 0.17%. However, note that Kamiya et al. (2018), with the largest sample (351 eyes followed for one year), and Alfonso et al. (2019), with the longest follow‐up (147 eyes, five years), did not report any cases. The low occurrence rate for this event confirms the safety of the lens.

Other complications have also been described. For example, rotation of the lens (>30°) was reported in several studies (Karandikar et al. 2015; Bhandari et al. 2016; Ganesh et al. 2017; Pjano et al. 2017; Kamiya et al. 2018); this event required re‐rotation or lens exchange surgery (eight eyes, representing 0.27%). A comparative study of rotational stability between the V4c versus V4 toric models concluded that both lenses have similar rotational stability (3.39° versus 4.17°, respectively; Hyun et al. 2017). Glare and haloes were reported in three studies, two of them by the same group (Karandikar et al. 2015; Bhandari et al. 2016), with high percentages (ranging from 23 to 27%, sample: 44 eyes), and another (Kamiya et al. 2018) with low values (from 2.4 %to 8.7%, sample: 351 eyes). Experimental and clinical studies reported that the central hole can be a source of glare and ring‐shaped dysphotopsia (Eppig et al. 2015; Eom et al. 2017). Disk halo size has been found to decrease significantly at one and three months after V4c ICL implantation (Chen et al. 2019), and that preoperative values were related to their SE, thus suggesting that the influence of myopia was removed after lens implantation (Zhao et al. 2018a). It was also found that the implantation of this lens did not induce a significant additional change in subjective intraocular forward scattering (Iijima et al. 2016), objective scatter (Huseynova et al. 2014) or quality of vision (Ferrer‐Blasco et al. .2013). Fernández‐Vega‐Cueto et al. (2018) and Kojima et al. (2018) reported several eyes that required ICL rotation (vertically) to decrease extremely high vault, similar to what has been described by other authors (Matarazzo et al. 2018). Pjano et al. (2017) reported one eye with retinal detachment three months after ICL surgery. However, this patient had high degenerative myopia and underwent prophylactic laser photocoagulation on both eyes before ICL implantation. Rizk et al. (2019) reported four eyes with pigment dispersion; they suggested that contact between the lens and the iris increases the probability of pigment dispersion. Studies with previous models of this ICL reported some cases (Fernandes et al. 2011), but only these cases of pigment dispersion with the V4c ICL have been published in the literature. Other events such as ICL exchange, iritis or touch‐up with a laser after the surgery happened infrequently (Pjano et al. 2017; Kamiya et al. 2018; Fernández‐Vega‐Cueto et al. 2018). In addition, in a couple of studies, this ICL model was implanted in eyes with peripheral primary iris and ciliary body cysts. They concluded that ICL implantation is a safe and effective procedure since the cysts did not change after the surgery and that their presence is not a contraindication for ICL surgery (Li et al. 2018; Zhao et al. 2018b).

Our analysis describes few adverse events related to ICL implantation, which support the safety of this lens when implanted for myopia correction in the short, medium and long term.

## Conclusions

The outcomes reported in the different studies analysed in the present review support the use of the V4c ICL model for myopia correction. More specifically, it provides patients with good visual acuity and good refractive outcomes, thus confirming the safety, efficacy and predictability of the procedure. Postoperative assessment of parameters such as IOP, ECD, ECD loss and vault confirms that their values remain generally adequate, even during long follow‐up, and that this lens appears to be safe as it removes the need to perform preoperative laser iridotomies or intraoperative iridectomies. We have to point out that few studies reported adverse events or complications, but in those that did, the prevalence was low. In our opinion, the lens design with its central hole, adequate patient selection and an accurate measurement of all the parameters that are required to calculate ICL size and power are all extremely important to achieve good postoperative outcomes in the short, medium and long term. Also, we consider that it is a shame that studies focusing on the outcomes of an ICL surgery do not include parameters as important as the ICL characteristics or others such as WTW or ACD, which are required to calculate the size and the power of the ICL or the safety of the surgery. It is clear that these parameters were indeed measured and used for the surgery plan; their publication would facilitate comparison between studies. We believe and recommend that these values should be included in any publication on ICL implantation procedures.
